# Spatial Metabolomics Reveals the Common and Compound-Specific Pharmacological Mechanisms of Two Alkaloids Against Infarcted Myocardium

**DOI:** 10.3390/ijms27167484

**Published:** 2026-08-21

**Authors:** Zixuan Zhang, Yixuan Lin, Feng Gao, Na Zhang, Jingyi Jiao, Huoli Yin, Tianzhen Liang, Herong Cui, Dong Bai, Haimin Lei

**Affiliations:** 1 School of Chinese Medicine, Beijing University of Chinese Medicine, Beijing 102401, China; zhangzx0719@163.com (Z.Z.); xiawubuchi@163.com (Y.L.); 18003250768@163.com (J.J.); 18811203987@163.com (H.Y.); polarisa1225@163.com (T.L.); 2 State Key Laboratory for Quality Ensurance and Sustainable Use of Dao-Di Herbs, Institute of Chinese Materia Medica, China Academy of Chinese Medical Sciences, Beijing 100700, China; fgao@icmm.ac.cn; 3 Institute of Basic Theory for Chinese Medicine, China Academy of Chinese Medical Sciences, Beijing 100700, China; bucmzn@163.com (N.Z.); baidong2000@126.com (D.B.); 4 School of Life Sciences, Beijing University of Chinese Medicine, Beijing 102401, China

**Keywords:** acute myocardial infarction, tetrahydropalmatine, berberine, multi-omics, lipid metabolism, spatial metabolomics

## Abstract

Acute myocardial infarction (AMI) is a leading cause of death worldwide, characterised by systemic inflammation and metabolic disorders. Tetrahydropalmatine (THP) and berberine (BBR) are major alkaloids derived from *Corydalis yanhusuo* and *Coptis chinensis*, respectively, both of which have been shown to be cardioprotective; however, whether their mechanisms differ remains unclear. In this study, we systematically compared THP and BBR in treating AMI using integrated spatial metabolomics (AFADESI-MSI), untargeted metabolomics, lipidomics, and molecular biology. The results showed that both compounds improved cardiac function, reduced fibrosis, and suppressed inflammation. Multi-omics revealed that although both regulate glycerophospholipid metabolism, their pathway preferences and functional roles diverge: THP primarily affects linoleic acid and acetylcholine metabolism with a greater propensity to restore membrane structural integrity, whereas BBR targets ether phospholipids and sphingolipids with preferential anti-inflammatory lipid modulation. At the enzymatic level, both downregulated CHKα, PEMT, ChAT, and PDHA1. A key difference is that THP uniquely upregulated acetylcholinesterase (AChE) mRNA expression, an effect absent with BBR. Spatial metabolomics directly visualised that both compounds reverse the accumulation of pro-inflammatory lysophosphatidylcholines (LPCs) and restore structural phosphatidylcholines (PCs) in the infarct region, thereby re-establishing regional lipid homeostasis. To our knowledge, this is the first integrated multi-omics comparison to suggest shared and distinct mechanisms of THP and BBR in AMI. Notably, the differential regulation of AChE, as visualised by spatial omics, may serve as a molecular basis for understanding their distinct therapeutic features, although further validation at the protein and enzymatic activity levels is warranted.

## 1. Introduction

Acute myocardial infarction (AMI) is a common cardiovascular emergency defined as ischaemic necrosis of myocardial tissue resulting from acute coronary artery occlusion. Its pathogenesis involves multiple intertwined pathophysiological processes, including exacerbated oxidative stress, heightened inflammatory responses, and abnormal ventricular remodelling [1]. Even when reperfusion therapy rapidly reopens the infarct-related artery and restores myocardial blood flow, subsequent ischaemia-reperfusion injury and long-term myocardial fibrosis continue to have a major impact on patients’ long-term quality of life and prognosis [2]. Owing to their multi-target and holistic regulatory properties, traditional Chinese medicine (TCM) and its active constituents have shown unique potential for the prevention and treatment of cardiovascular diseases in recent years.

*Coptis chinensis* and *Corydalis yanhusuo* are frequently used in combination in TCM formulas for the treatment of cardiovascular diseases and chest pain. The primary pharmacologically active constituents of both herbs are protoberberine-type alkaloids. Berberine (BBR), the main active constituent of C. chinensis, has been shown to reduce cardiomyocyte apoptosis via the AMPK/mTOR signalling pathway [3,4,5], whereas tetrahydropalmatine (THP), the active constituent of C. yanhusuo, exerts significant anti-inflammatory effects by inhibiting the TLR4/NF-κB pathway [6,7,8]. However, systematic comparisons of the overall efficacy differences between these two herbs and their “component–effect” relationships remain insufficient, especially regarding a thorough elucidation of the molecular mechanisms underlying the phenomenon of “treating the same disease with different approaches.” Instead, current research primarily focuses on the mechanisms of individual components.

With the advancement of metabolomics and lipidomics technologies, the multi-component, multi-target mechanisms of action of TCM have been elucidated in greater detail. In particular, spatial metabolomics technology (e.g., AFADESI-MSI) can visually display the regional distribution characteristics of metabolites within the tissue microenvironment, offering new insights into the patterns of drug-induced metabolic regulation at the lesion site [9]. Against this backdrop, and to systematically investigate the effects of THP and BBR on lipid metabolic dynamics and acetylcholine pathways in an AMI model, this study combines AFADESI-MSI spatial metabolomics, untargeted metabolomics, lipidomics, and molecular biology techniques. From both the holistic and spatial dimensions of metabolic networks, the aim is to clarify the mechanisms underlying “treating the same disease with different approaches” and the contemporary scientific implications of these mechanisms. In particular, we sought to determine whether these two alkaloids exert compound-specific functional preferences, such as differential regulation of inflammatory lipid species versus structural membrane lipids, which may underlie their distinct therapeutic profiles.

## 2. Results

### 2.1. THP and BBR Improve Cardiac Function in AMI Mice

**Figure 1:**
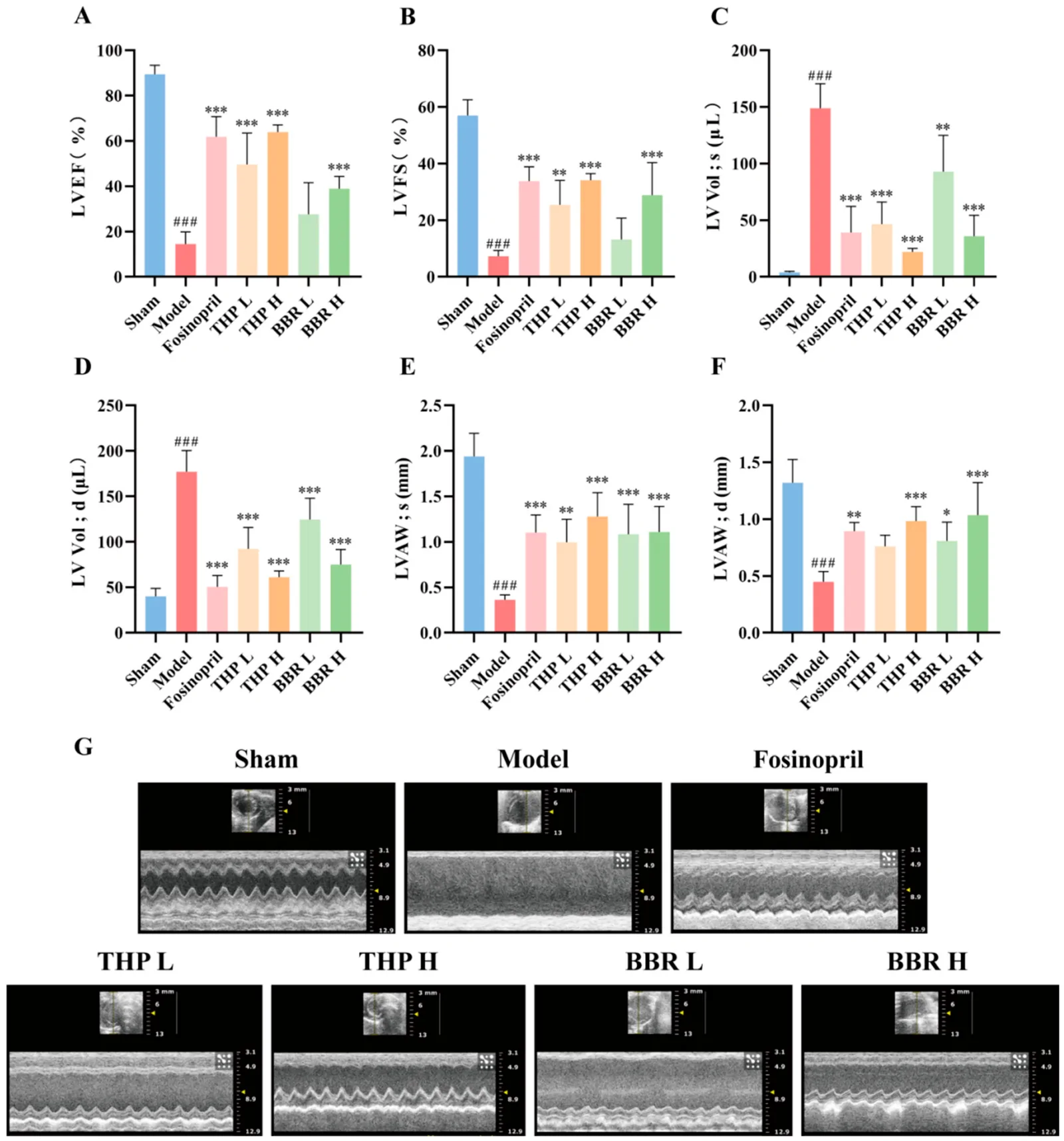
Echocardiography. (**A**) LVEF. (**B**) LVFS. (**C**) LVVol; s. (**D**) LVVol; d. (**E**) LVAW; s. (**F**) LVAW; d. (**G**) Cardiac ultrasound images. Compared to the sham-operated group, ^###^ *p* < 0.001; compared to the model group, * *p* < 0.05, ** *p* < 0.01, *** *p* < 0.001.

Echocardiography revealed that AMI mice (model group) exhibited significantly reduced LVEF and LVFS compared with the sham group (*p* < 0.001), indicating severe cardiac dysfunction (Figure 1A,B). Treatment with fosinopril, THP H, and BBR H significantly improved LVEF and LVFS (*p* < 0.001 vs. model). LVVol;d and LVVol;s were markedly increased in the model group, reflecting ventricular dilatation, and were reduced by drug treatments (*p* < 0.001, Figure 1C,D). LVAW;d and LVAW;s were decreased in model mice and partially restored by THP and BBR (*p* < 0.05–0.001, Figure 1E,F). These findings are consistent with previous studies [10], indicating that both high-dose THP and BBR effectively protect cardiac function following myocardial infarction.

### 2.2. THP and BBR Reduce Myocardial Injury and Fibrosis

**Figure 2:**
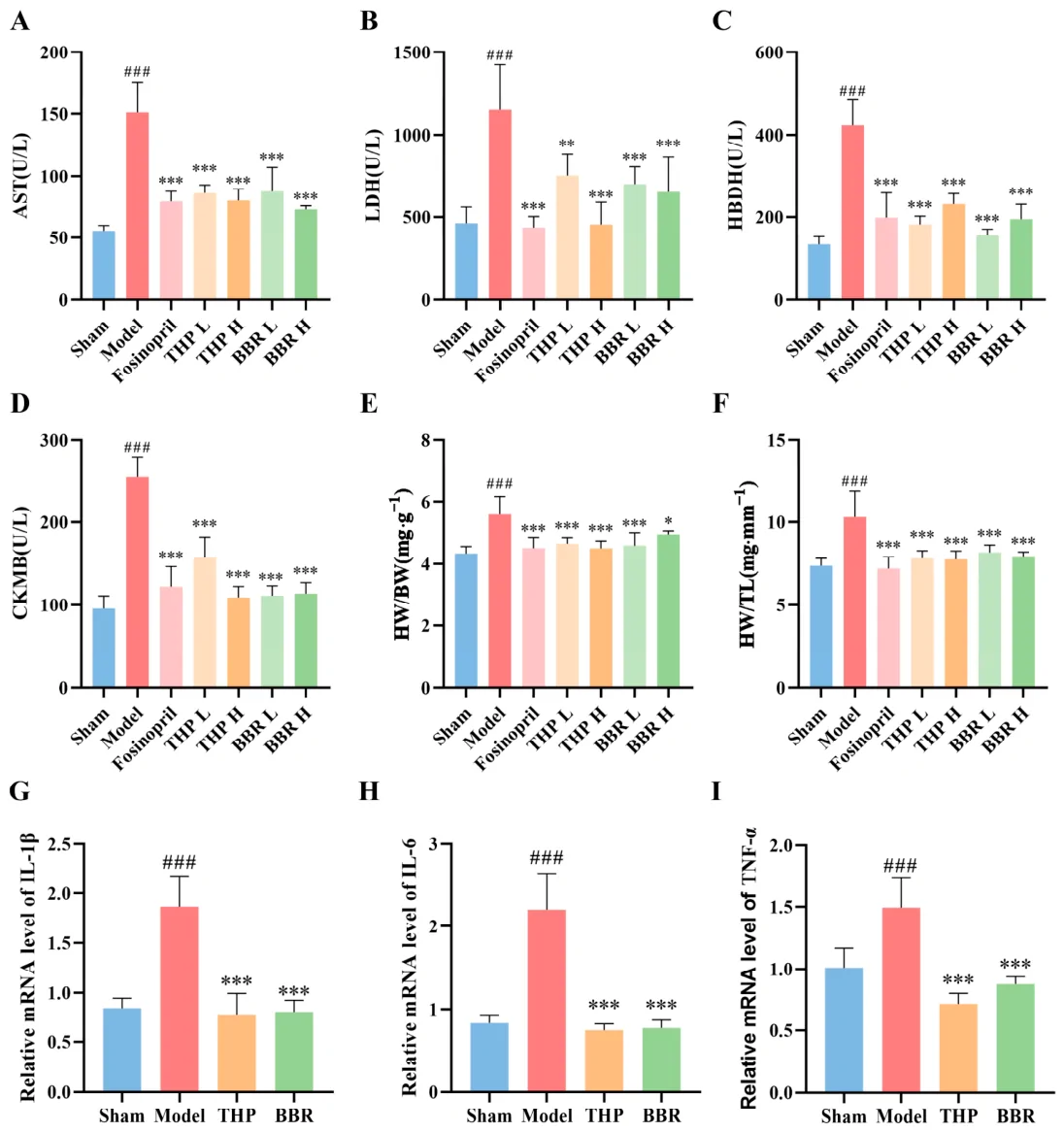
Serum myocardial enzyme profile expression levels. (**A**) AST. (**B**) LDH. (**C**) HBDH. (**D**) CK-MB. (**E**) HW/BW. (**F**) HW/TL. (**G**) IL-1β. (**H**) IL-6. (**I**) TNF-α. Compared to the sham-operated group, ^###^ *p* < 0.001; compared to the model group, * *p* < 0.05, ** *p* < 0.01, *** *p* < 0.001.

**Figure 3:**
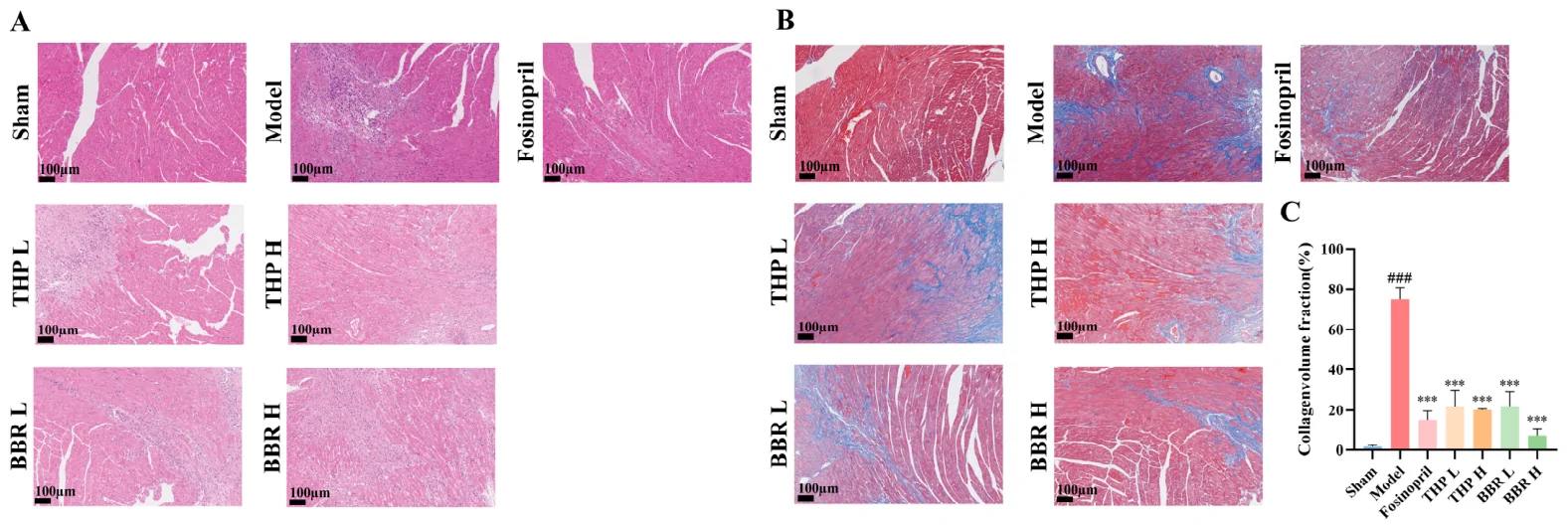
HE and Masson staining of heart tissue from each group of mice. (**A**) HE staining (200×). (**B**) Masson staining (200×). (**C**) Collagen volume fraction. Compared to the sham-operated group, ^###^ *p* < 0.001; compared to the model group, *** *p* < 0.001.

Serum levels of AST, LDH, HBDH, CK-MB and CK were significantly elevated in model mice, indicating severe myocardial damage (Figure 2A–D). The serum CK levels are shown in Figure S1. All drug treatments reduced these enzyme activities, with THP H and BBR H showing comparable efficacy to fosinopril. Direct statistical comparison between THP H and BBR H groups revealed no significant differences in serum cardiac enzyme levels. Heart weight indices (HW/BW and HW/TL) were increased in model group mice and significantly reduced by drug treatments (Figure 2E,F). H&E staining showed disorganized cardiomyocytes, nuclear condensation, and inflammatory infiltration in the infarct zone of model mice, which were ameliorated by THP and BBR (Figure 3A). Masson trichrome staining revealed extensive collagen deposition in the model group (collagen volume fraction ~75%), which was markedly reduced by drug treatments (*p* < 0.001, Figure 3B,C). Direct statistical comparison between THP and BBR groups showed no significant differences in heart weight indices, histopathological improvements, or collagen volume fraction.

### 2.3. THP and BBR Suppress Myocardial Inflammation

**Figure 4:**
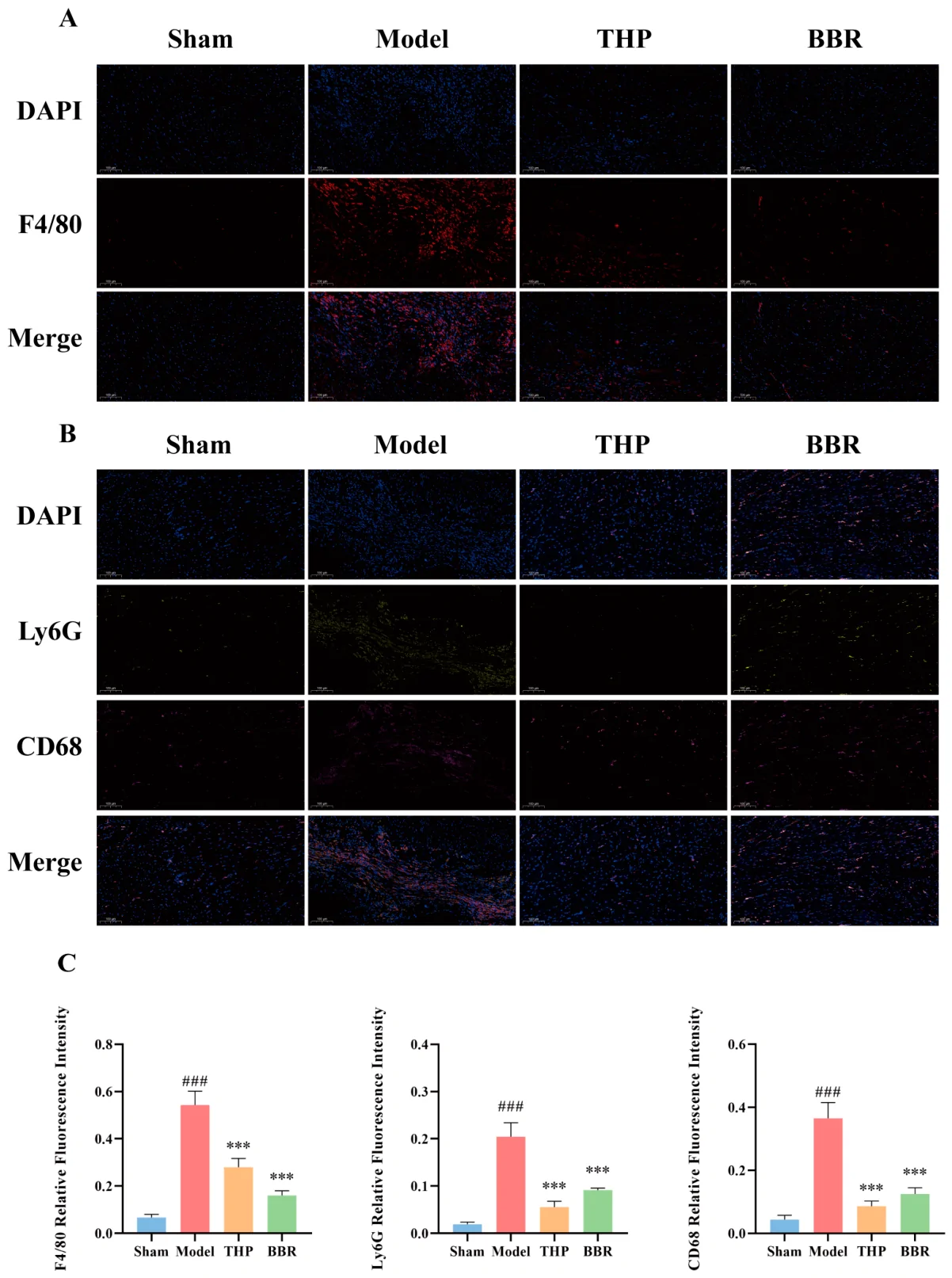
Immunofluorescence staining of F4/80 (red), Ly6G (yellow), and CD68 (pink) in myocardial tissue and statistical analysis of fluorescence intensity (**A**–**C**). Compared to the sham-operated group, ^###^ *p* < 0.001; compared to the model group, *** *p* < 0.001.

Since both compounds exhibited dose-dependent effects in the preliminary screening, the comparisons in this section and Section 2.6 and Section 2.7 below focus primarily on the high-dose groups (THP H, 60 mg/kg; BBR H, 60 mg/kg). mRNA expression of pro-inflammatory cytokines IL-1β, IL-6, and TNF-α was upregulated in the model group and significantly downregulated by THP and BBR (*p* < 0.001, Figure 2G–I). Immunofluorescence staining showed increased infiltration of F4/80+ macrophages, CD68+ macrophages, and Ly6G+ neutrophils in the infarct zone of model mice, which was attenuated by both drugs (*p* < 0.001, Figure 4).

### 2.4. Metabolomics Reveals Shared and Distinct Metabolic Pathways

**Figure 5:**
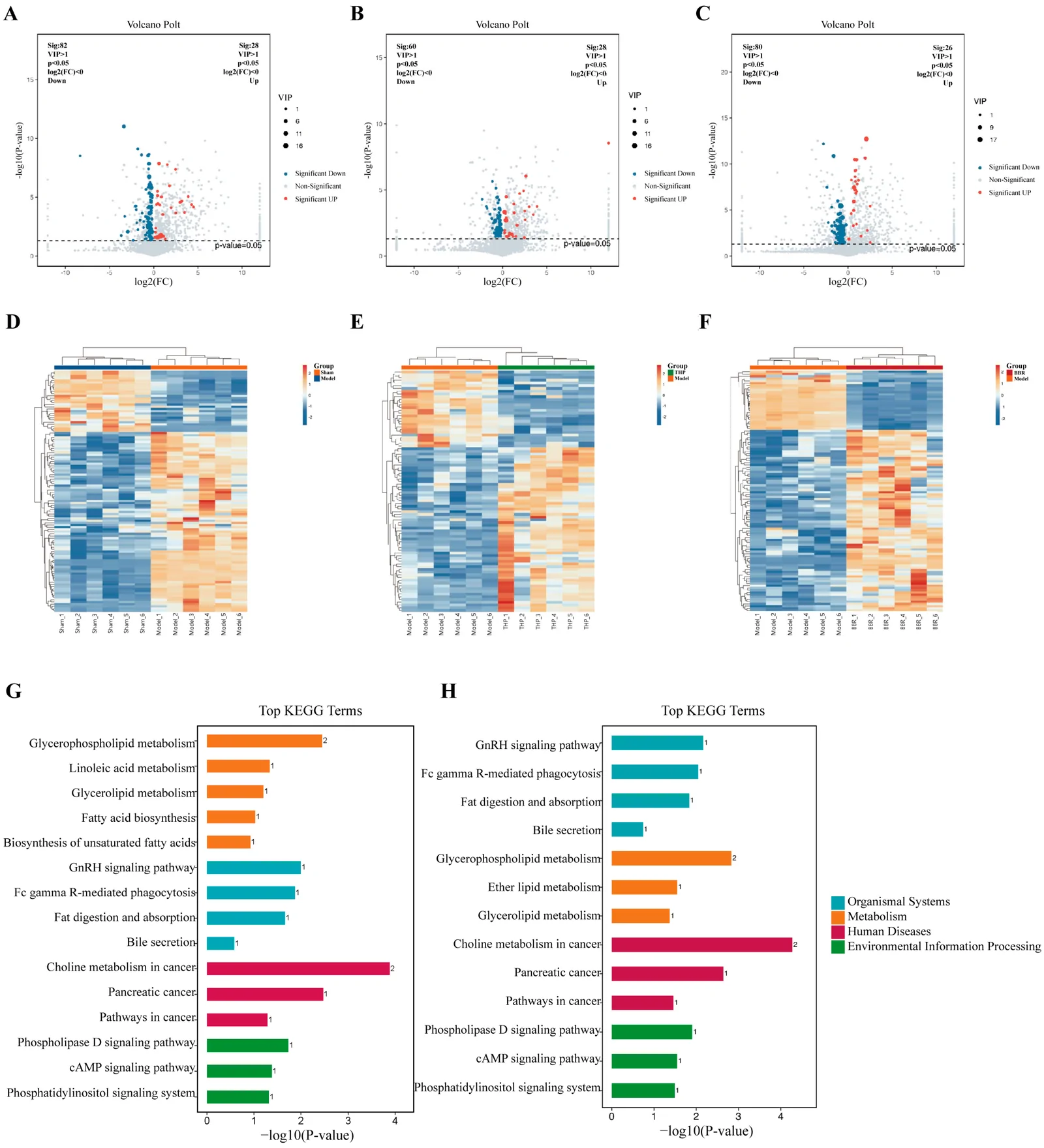
Untargeted serum metabolomics. (**A**) Volcano plot (Sham vs. Model). (**B**) Volcano plot (THP vs. Model). (**C**) Volcano plot (BBR vs Model). (**D**) Cluster heatmap (Sham vs. Model). (**E**) Cluster heatmap (THP vs. Model). (**F**) Cluster heatmap (BBR vs. Model). (**G**) KEGG enrichment of differential metabolites between THP and Model groups. (**H**) KEGG enrichment of differential metabolites between BBR and Model groups.

Untargeted metabolomics of serum identified 3138 metabolites. OPLS-DA analysis showed clear separation between groups (Figure S2). Compared to sham, the model group had 110 differential metabolites (82 down, 28 up). THP treatment reversed 88 metabolites (60 down, 28 up), while BBR reversed 106 metabolites (80 down, 26 up) (Figure 5A–F). KEGG enrichment indicated that THP mainly affected glycerophospholipid metabolism, linoleic acid metabolism, and fatty acid biosynthesis [11] (Figure 5G), whereas BBR primarily modulated glycerophospholipid metabolism, ether lipid metabolism, and glycerolipid metabolism (Figure 5H).

### 2.5. Lipidomics Shows Modulation of Phospholipid Metabolism

**Figure 6:**
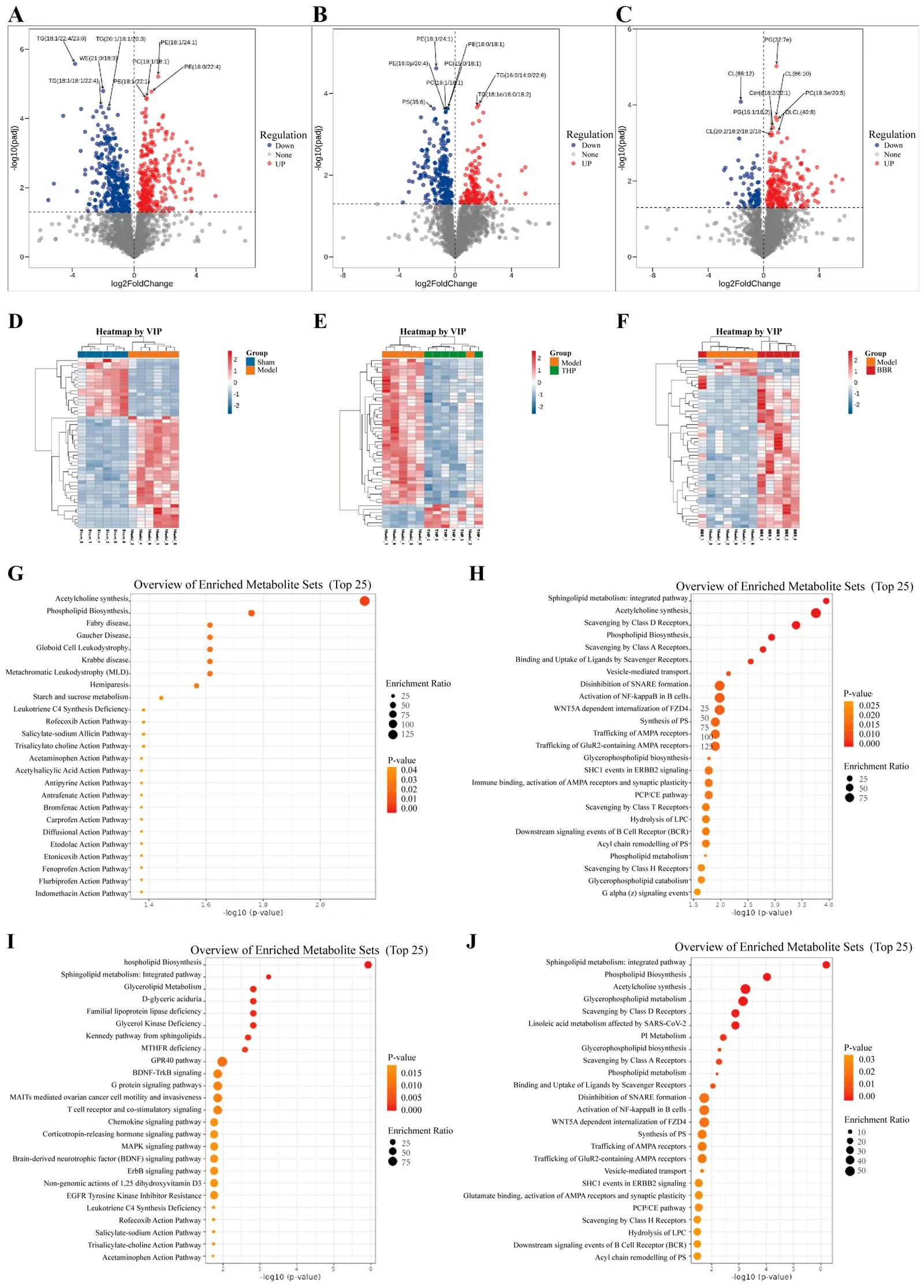
Cardiac lipidomics. (**A**) Volcano plot (Sham vs. Model). (**B**) Volcano plot (THP vs. Model). (**C**) Volcano plot (BBR vs. Model). (**D**) Cluster heatmap (Sham vs. Model). (**E**) Cluster heatmap (THP vs. Model). (**F**) Cluster heatmap (BBR vs. Model). (**G**) KEGG enrichment of differential metabolites between THP and Model groups (positive ion mode). (**H**) KEGG enrichment of differential metabolites between THP and Model groups (negative ion mode). (**I**) KEGG enrichment of differential metabolites between BBR and Model groups (positive ion mode). (**J**) KEGG enrichment of differential metabolites between BBR and Model groups (negative ion mode).

Cardiac lipidomics identified 621 differential lipids between model and sham (334 down, 287 up). THP reversed 381 lipids (223 down, 148 up), and BBR reversed 303 lipids (68 down, 235 up) (Figure 6A–C). OPLS-DA score plots confirmed robust separation between groups (Figure S3). Pathway enrichment revealed that both drugs modulated glycerophospholipid metabolism and sphingolipid metabolism [12] (Figure 6G–J). Notably, THP enriched acetylcholine synthesis pathways, whereas BBR enriched ether lipid metabolism.

### 2.6. THP and BBR Regulate Phospholipid and Acetylcholine Metabolic Enzymes

**Figure 7:**
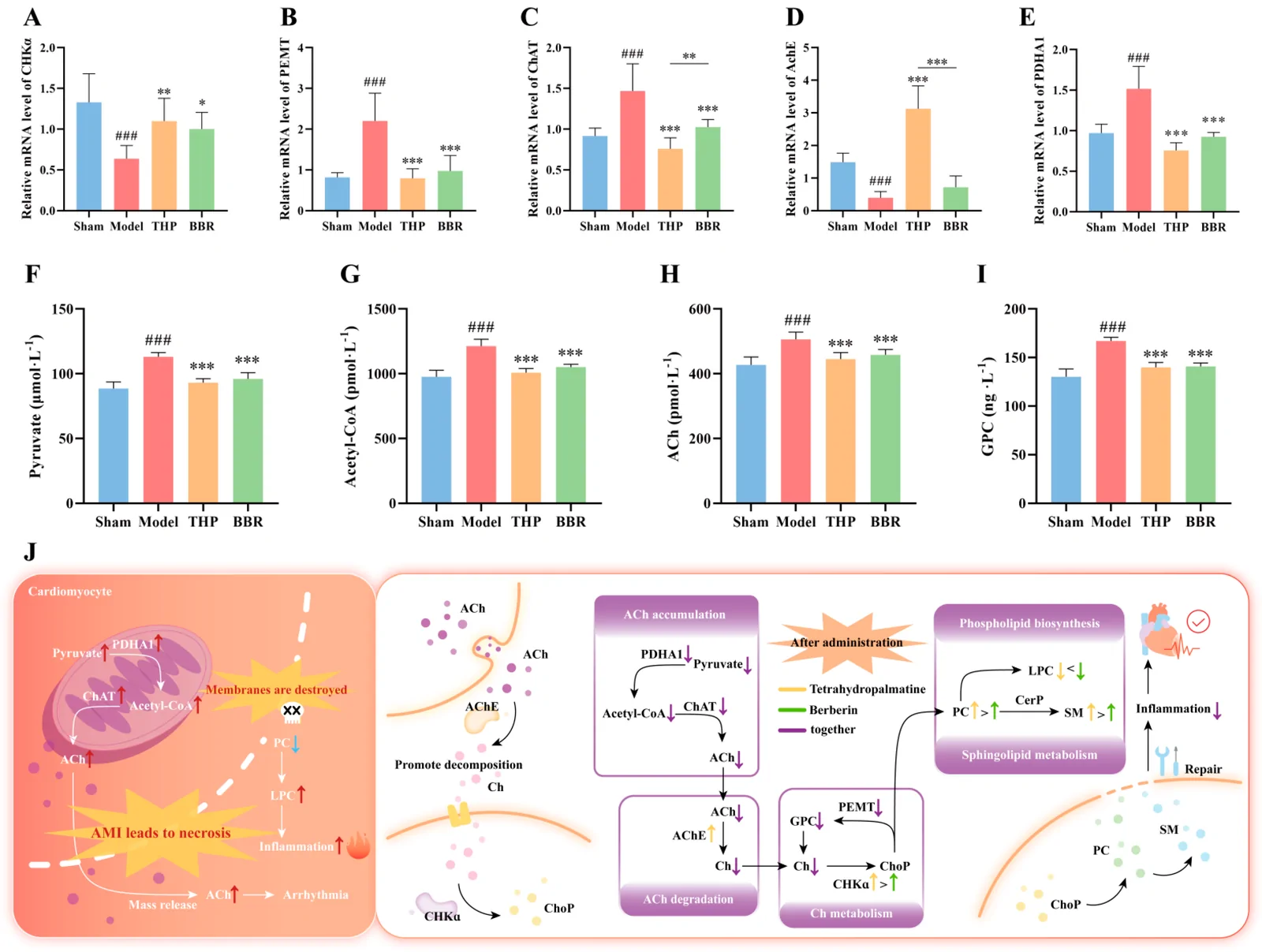
RT-PCR determination of metabolic enzyme mRNA expression levels and ELISA quantification of metabolite content. (**A**) CHKα. (**B**) PEMT. (**C**) ChAT. (**D**) AChE. (**E**) PDHA1. (**F**) Pyruvate. (**G**) Acetyl-CoA. (**H**) ACh. (**I**) GPC. (**J**) Mechanism schematic. Compared to the sham-operated group, ^###^ *p* < 0.001; compared to the model group, * *p* < 0.05, ** *p* < 0.01, *** *p* < 0.001.

RT-PCR showed that mRNA levels of CHKα were significantly decreased, whereas those of PEMT, ChAT, and PDHA1 were significantly elevated in the model group (*p* < 0.001). Treatment with THP and BBR significantly reversed these changes (*p* < 0.05 or *p* < 0.001) (Figure 7A–C,E). For AChE, model mice exhibited decreased mRNA expression; THP significantly upregulated AChE mRNA to levels even above those of the sham group (*p* < 0.001), whereas BBR only restored it to near-sham levels, showing no significant difference from the model group (Figure 7D). Consistently, metabolite levels of pyruvate, acetyl-CoA, acetylcholine, and glycerophosphocholine were increased in model mice and significantly reduced by THP and BBR treatments (*p* < 0.001, Figure 7F–I). Direct statistical comparison between THP and BBR groups revealed a significant difference in ChAT expression (*p* < 0.01) and AChE expression (*p* < 0.001), but not in CHKα, PEMT, PDHA1, or any of the metabolites.

### 2.7. Spatial Metabolomics Reveals Restoration of Lipid Homeostasis in Infarct Zone

**Figure 8:**
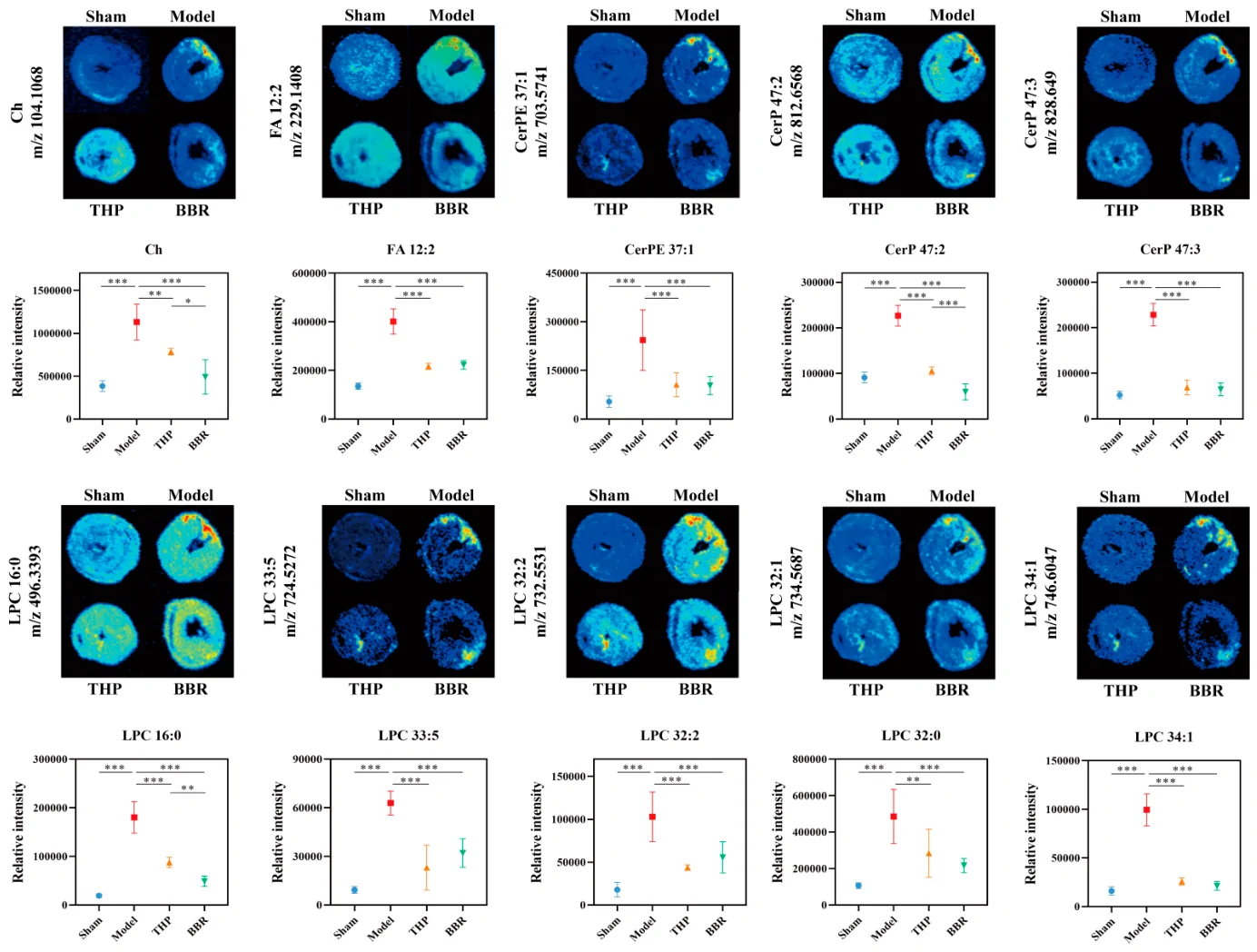
Distribution imaging map of lipid metabolites showing an accumulation trend in the AMI infarct region. * *p* < 0.05, ** *p* < 0.01, *** *p* < 0.001.

**Figure 9:**
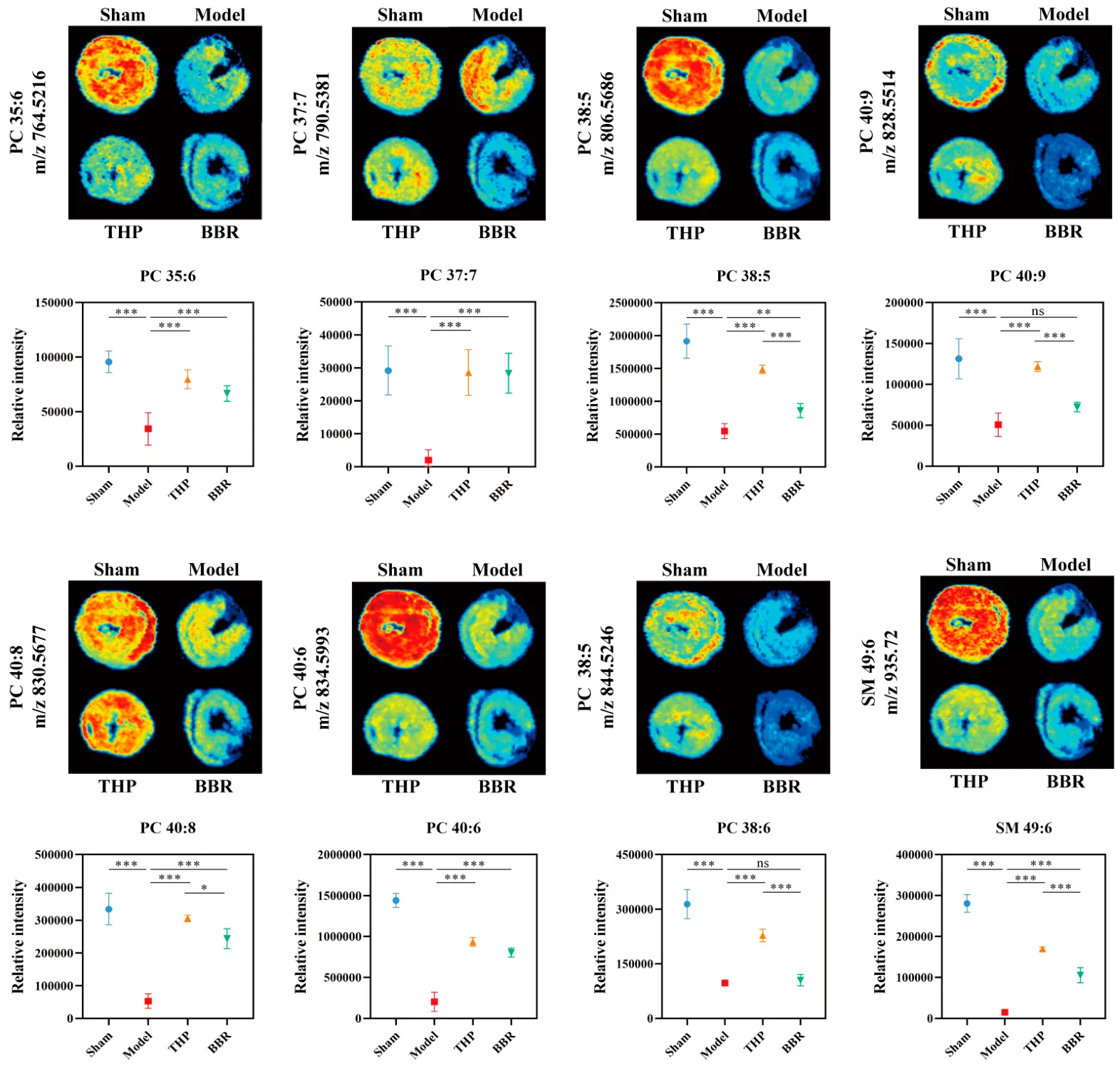
Distribution imaging map of lipid metabolites showing a depletion trend in the AMI infarct region. * *p* < 0.05, ** *p* < 0.01, *** *p* < 0.001.

AFADESI-MSI imaging showed that in the infarct region of model mice, pro-inflammatory LPC species (e.g., LPC 16:0, LPC 18:1) accumulated, while structural PC species (e.g., PC 34:1, PC 36:2) were depleted (Figure 8 and Figure 9). Both THP and BBR treatments reversed these changes, reducing LPCs and restoring PCs in the infarct zone. Additionally, sphingomyelins (SM 34:1) [13] were elevated in the model and reduced by drugs, while ceramide phosphoethanolamines (CerPE 38:1) were decreased in the model and restored by treatment. Notably, BBR treatment resulted in a greater reduction than THP in Ch, FA 12:2, CerP 47:2, and LPC 16:0 (*p* < 0.01 or *p* < 0.001), whereas THP treatment achieved greater upregulation than BBR in PC 38:5, PC 40:9, PC 40:8, PC 38:6, and SM 49:6 (*p* < 0.05 or *p* < 0.001), suggesting compound-specific lipid modulation in the infarct region. Collectively, these differential lipidomic profiles suggest that BBR may preferentially alleviate inflammation by more effectively reducing pro-inflammatory lipid species, whereas THP may exhibit a greater propensity to restore membrane structural integrity by upregulating structural PCs and sphingomyelins. These spatial data confirm that THP and BBR locally rebalance lipid metabolism in injured myocardium. PCA and OPLS-DA analyses clearly distinguished the infarct zone from the non-infarct zone (Figure S4).

## 3. Discussion

In this study, we comprehensively evaluated the cardioprotective effects of THP and BBR in a mouse model of AMI using an integrated multi-omics approach. Consistent with previous reports [3,4,6,10], both compounds improved cardiac function, reduced myocardial injury and fibrosis, and suppressed inflammatory responses. However, whereas previous studies focused on individual molecular pathways, our integrated multi-omics approach provides evidence that both drugs converge on the regulation of glycerophospholipid and acetylcholine metabolism, yet exhibit distinct pathway preferences and differential enzymatic regulation, particularly with respect to AChE.

The improvement in cardiac function observed by echocardiography (increased LVEF, LVFS, and wall thickness; reduced ventricular volumes) aligns with earlier work showing that THP and BBR can preserve systolic and diastolic function post-infarction [14,15]. The reduction in serum cardiac enzymes (AST, LDH, HBDH, CK-MB) and heart weight indices further confirms their ability to limit myocardial damage and adverse remodelling, as similarly reported for angiotensin-converting enzyme inhibitors such as fosinopril [16,17]. Histopathological examination revealed that THP and BBR ameliorated cardiomyocyte disarray, inflammatory infiltration, and collagen deposition, corroborating their anti-fibrotic and anti-inflammatory actions [18,19,20].

Immunofluorescence and cytokine mRNA data demonstrated that both drugs suppressed macrophage and neutrophil infiltration and downregulated the expression of pro-inflammatory cytokines IL-1β, IL-6, and TNF-α. According to previous studies, these anti-inflammatory effects may be mediated through inhibition of the TLR4/NF-κB pathway by THP [7,8] and modulation of AMPK/mTOR or the NLRP3 inflammasome by BBR [5,21,22]. Both compounds limited the recruitment of inflammatory cells into the infarct zone, which is consistent with the reduction in pro-inflammatory LPCs observed in spatial metabolomics, as LPCs are known chemoattractants and activators of immune cells [23,24,25].

Metabolomics and lipidomics revealed that both THP and BBR regulate glycerophospholipid metabolism, but with distinct preferences: THP primarily affects linoleic acid metabolism and acetylcholine synthesis, whereas BBR targets ether-phospholipids and sphingolipids. This divergence may reflect differences in their chemical structures and molecular targets [26,27]. At the enzymatic level, both drugs downregulated CHKα, PEMT, ChAT, and PDHA1, leading to reduced levels of pyruvate, acetyl-CoA, acetylcholine, and glycerophosphocholine, a coordinated suppression likely representing a compensatory response to ischaemia-induced metabolic overactivation [28]. However, a striking difference was observed in AChE regulation: THP significantly upregulated AChE mRNA, whereas BBR only restored it to baseline levels. Increased AChE expression by THP may accelerate acetylcholine breakdown, limiting cholinergic overstimulation and potentially contributing to its anti-inflammatory effects [29,30]. In contrast, BBR may rely more on alternative pathways such as AMPK activation to achieve cardioprotection [3]. This enzymatic distinction positions AChE as a molecular discriminant between the two compounds.

Spatial metabolomics provided a novel dimension by visualising lipid alterations directly within the infarct zone. Both drugs reversed the accumulation of pro-inflammatory LPCs and restored structural PCs and CerPEs, confirming that they locally rebalance lipid metabolism. LPCs are generated by ischaemia-activated phospholipase A2 [31] and are potent pro-inflammatory mediators; their reduction by THP and BBR correlates with the decreased inflammatory infiltration seen histologically. Conversely, PCs are essential for membrane stability [32,33], and their recovery likely preserves cardiomyocyte integrity. Beyond this common mechanism, our spatial metabolomic data further revealed compound-specific functional preferences: BBR more effectively reduced pro-inflammatory lipid species (e.g., LPC 16:0, CerP 47:2), whereas THP more prominently restored structural membrane lipids (e.g., PC 38:5, PC 40:9, SM 49:6), supporting a functional divergence wherein BBR preferentially targets inflammation while THP exhibits a greater propensity for restoring membrane integrity.

While our findings demonstrate the cardioprotective effects of THP and BBR in a preclinical AMI model, it is worth considering how these compounds might integrate with current standard-of-care therapies for AMI. Contemporary management of AMI relies on rapid revascularisation, dual antiplatelet therapy, statins, angiotensin-converting enzyme inhibitors (ACEIs) or angiotensin receptor blockers (ARBs), and β-blockers, which together have substantially improved outcomes by restoring coronary flow, stabilising plaques, and mitigating neurohormonal activation [16,17]. However, even with optimal pharmacotherapy, secondary pathological processes including persistent low-grade inflammation and metabolic dysregulation continue to drive adverse ventricular remodelling and heart failure progression [18,19,20,28]. In this context, THP and BBR may offer complementary therapeutic value by targeting these residual risks. Both compounds suppress inflammatory responses and modulate lipid metabolism through pathways that are not directly addressed by current standard therapies, suggesting that they could serve as adjunctive agents to mitigate post-infarction inflammation and metabolic imbalance. Several lines of evidence support this possibility: BBR has been shown to activate AMPK and improve glucose and lipid metabolism [3,4], whereas THP exerts anti-inflammatory effects through TLR4/NF-κB inhibition [7,8], mechanisms that could synergise with statins or ACEIs. However, several limitations must be acknowledged before clinical translation. First, the oral bioavailability of both BBR and THP is relatively low due to extensive first-pass metabolism and poor intestinal absorption, which may limit their systemic exposure and necessitate careful consideration of dosing strategies or formulation optimisation [26]. Second, the doses used in this study (30–60 mg/kg in mice) may not directly translate to human equivalents, and the safety profile of long-term use, particularly in combination with standard therapies, requires systematic evaluation. Third, our findings are derived from a single animal model and may not fully recapitulate the complexity of human AMI, particularly in patients with comorbidities such as diabetes or hypertension. Therefore, while THP and BBR show promise as complementary candidates targeting inflammation and metabolic dysregulation, rigorous pharmacokinetic, toxicological, and clinical studies are needed to establish their role alongside evidence-based therapies.

Future translational studies should explore the functional consequences of AChE upregulation by THP and investigate whether combination therapy with standard-of-care agents could yield additive or synergistic benefits. In addition, the translational potential of these findings warrants further investigation through pharmacokinetic and toxicological evaluations, as well as well-designed clinical trials to assess efficacy and safety in patients with AMI.

## 4. Materials and Methods

### 4.1. Animals and AMI Model

Male C57BL/6J mice (8–10 weeks old, 22–25 g) were obtained from Beijing Speifu Biotechnology Co., Ltd. (China). All animal experiments were strictly conducted in accordance with the requirements of animal ethical review and approved by the Animal Ethics Committee of Beijing University of Chinese Medicine (Approval No. BUCM20250701-003), complying with the National Regulations for the Administration of Laboratory Animals. Mice were housed under specific pathogen-free conditions at 22–24 °C with a 12 h light/dark cycle and had free access to standard chow and water.

After 3 days of acclimatization, acute myocardial infarction (AMI) was induced by permanent ligation of the left anterior descending (LAD) coronary artery as previously described [34]. Briefly, mice were anesthetized with tribromoethanol (250 mg/kg, i.p.), intubated, and mechanically ventilated. A left thoracotomy was performed at the third to fourth intercostal space, the pericardium was gently removed, and the LAD was ligated with a 7-0 silk suture. Sham-operated mice underwent the same surgical procedure without LAD ligation. Successful ligation was confirmed by echocardiography performed 24 h post-surgery. After surgery, all mice received penicillin (40,000 U/mouse, i.m.) to prevent infection and were placed on a warming pad (37 °C) until recovery from anesthesia.

Twenty-four hours after surgery, surviving mice were randomly divided into seven groups (n = 6 per group): Sham, Model, Fosinopril (10 mg/kg), BBR low-dose (30 mg/kg), BBR high-dose (60 mg/kg), THP low-dose (30 mg/kg), and THP high-dose (60 mg/kg). All drugs were suspended in 0.5% sodium carboxymethyl cellulose (CMC-Na) and administered once daily by gavage for 7 consecutive days, starting 24 h post-AMI. The Sham and Model groups received an equal volume of 0.5% CMC-Na vehicle.

### 4.2. Echocardiography

After 7 days of drug administration, mice were anesthetized with isoflurane gas anesthesia in the supine position. Heart rate was maintained at 500–550 beats/min throughout the procedure. Transthoracic echocardiography was performed using a Vevo 2100 imaging system equipped with an MS-400 transducer (VisualSonics, Toronto, ON, Canada). The probe was placed along the short axis of the heart to obtain the short-axis view under B-mode, and the probe angle was finely adjusted until the left ventricle and papillary muscles were clearly visible in the acquired images. Under M-mode tracing, the measurement cursor was placed perpendicular to the interventricular septum and left ventricular posterior wall at the level of the papillary muscles, intersecting the maximal left ventricular diameter. Left ventricular ejection fraction (LVEF), left ventricular fractional shortening (LVFS), left ventricular end-diastolic volume (LVVol;d), left ventricular end-systolic volume (LVVol;s), left ventricular anterior wall thickness at end-systole (LVAW;s), and left ventricular anterior wall thickness at end-diastole (LVAW;d) were measured. All parameters were derived from stable waveforms of three consecutive cardiac cycles, and data were processed using the three-cycle averaging method.

### 4.3. Serum Biochemistry

After echocardiography, mice were anesthetized with tribromoethanol (250 mg/kg, i.p.), and blood samples were collected from the abdominal aorta. The samples were allowed to stand at room temperature for 2 h and then centrifuged at 4 °C, 3000 rpm for 15 min in a high-speed refrigerated centrifuge. The upper serum layer was carefully aspirated and stored at −80 °C until analysis. After thawing at 4 °C, serum levels of creatine kinase (CK), α-hydroxybutyrate dehydrogenase (α-HBDH), aspartate aminotransferase (AST), lactate dehydrogenase (LDH), and creatine kinase-MB isoenzyme (CK-MB) were measured using an automatic biochemical analyzer (AU480, Beckman Coulter, Brea, CA, USA).

### 4.4. Histopathology

Preparation of paraffin-embedded sections: Excised mouse hearts were fixed in 4% paraformaldehyde at 4 °C for 48 h and then rinsed with normal saline and dehydrated through graded ethanol (70% → 100%) using an automatic tissue dehydrator, with each step lasting 1–2 h. Tissues were cleared in xylene for 2 h, embedded in paraffin, and sectioned at 4 μm thickness.

Staining and observation: Sections were deparaffinized, rehydrated through graded ethanol, and stained with hematoxylin and eosin (H&E) for morphological evaluation or Masson’s trichrome for collagen visualization. Whole-slide digital images were acquired using a digital pathology scanning system to assess cardiomyocyte arrangement, inflammatory infiltration, and fibrosis. Collagen volume fraction (CVF) was quantified using Fiji (ImageJ 1.53c), calculated as the ratio of blue-stained area to total tissue area from five randomly selected fields per section.

### 4.5. Immunofluorescence

Paraffin-embedded heart sections were deparaffinized in xylene, rehydrated through graded ethanol, and subjected to antigen retrieval in citrate buffer (pH 6.0) using a microwave oven. After blocking with 3% BSA for 30 min at room temperature, sections were incubated overnight at 4 °C with primary antibodies against F4/80, CD68, and Ly6G (1:200, Abcam, Cambridge, UK), followed by Alexa Fluor-conjugated secondary antibodies (1:500, Invitrogen, Waltham, WA, USA) for 50 min at room temperature in the dark. Nuclei were counterstained with DAPI for 10 min. Images were captured using a fluorescence microscope (E100, Nikon, Tokyo, Japan), and fluorescence intensity was quantified using ImageJ from five randomly selected fields per section.

### 4.6. Untargeted Metabolomics of Serum

Serum samples (50 µL) were mixed with 200 µL methanol/acetonitrile (1:1, *v*/*v*) containing 4-chloro-DL-phenylalanine as an internal standard, vortexed for 2 min, and centrifuged at 12,000× g for 10 min at 4 °C. The supernatant was filtered through a 0.22 µm PVDF membrane and analyzed using a UHPLC-Q-Exactive Orbitrap MS system (Thermo Fisher, Waltham, WA, USA) in positive and negative ion modes. Chromatographic separation was performed on an ACQUITY UPLC HSS T3 column (2.1 × 100 mm, 1.8 µm) with a mobile phase of water (A) and acetonitrile (B), both containing 0.1% formic acid. Data were processed using Compound Discoverer 3.2, and metabolites were identified based on accurate mass, MS/MS fragmentation, and database matching (HMDB, KEGG, METLIN). Multivariate analysis, including PCA, PLS-DA, and OPLS-DA, was conducted using SIMCA 14.1 with 200-fold permutation tests. Differential metabolites were defined by VIP > 1 and *p* < 0.05. KEGG pathway enrichment was performed using MetaboAnalyst 5.0.

### 4.7. Lipidomics of Cardiac Tissue

Cardiac tissue (30 mg) was homogenized, and lipids were extracted with methanol (300 µL) containing internal standards, followed by the addition of methyl tert-butyl ether (1000 µL) and ultrapure water (300 µL) for phase separation. The organic phase was dried under nitrogen and reconstituted in 100 µL isopropanol/acetonitrile (9:1, *v*/*v*). Lipidomics analysis was performed on a UHPLC system coupled with a Q-Exactive Orbitrap MS using an ACQUITY Premier CSH C18 column (2.1 × 100 mm, 1.7 µm). The mobile phase consisted of acetonitrile/water (3:2, *v*/*v*) with 10 mM ammonium formate (A) and isopropanol/acetonitrile (9:1, *v*/*v*) with 10 mM ammonium formate (B). Mass spectrometry was performed in positive and negative ion modes with a spray voltage of 2.5 kV, capillary temperature of 325 °C, and auxiliary gas heating temperature of 300 °C. Data were acquired in full scan mode followed by data-dependent MS/MS (TopN = 10) with a collision energy of 30%. Data processing and statistical analysis were similar to metabolomics. Lipid species were identified using LipidSearch 4.2.

### 4.8. Spatial Metabolomics by AFADESI-MSI

Frozen heart tissues were embedded in OCT compound, sectioned at 10 μm thickness using a cryostat, and thaw-mounted onto glass slides. AFADESI-MSI analysis was performed in positive ion mode over a mass range of *m*/*z* 100–1000 with a mass resolution of 120,000. The tissue sections were continuously scanned at a constant rate of 0.2 mm·s^−1^ in the *x*-axis and 0.1 mm·s^−1^ in the *y*-axis direction. The ion source parameters were set as follows: spray voltage 3.0 kV, capillary temperature 350 °C, auxiliary nitrogen pressure 0.6 MPa, and transfer gas flow rate 45 L·min^−1^. The spray solvent was methanol containing 1% trifluoroacetic acid delivered at 4 μL·min^−1^. Raw data (.raw) were converted to .cdf format and processed using MassImager software 1.0 for image reconstruction, background subtraction, and data extraction. Peak picking, alignment, isotope removal, and normalization were performed using MarkViewTM software 1.2.1. Multivariate statistical analysis was conducted using SIMCA-P 14.1, and differential metabolites were selected based on VIP > 1, *p* < 0.05, and fold change >1.2 or <0.8. Metabolite identification was performed by accurate mass matching against Lipidmaps and HMDB databases, and pathway analysis was performed using MetaboAnalyst 5.0.

### 4.9. Real-Time PCR

Total RNA was extracted from cardiac tissue using TRIzol reagent (Invitrogen) according to the manufacturer’s instructions. RNA concentration and purity were determined using a nucleic acid ultraviolet spectrophotometer. cDNA was synthesized using PrimeScript RT Master Mix (Takara, Kyoto, Japan) with an initial incubation at 70 °C for 5 min, followed by 42 °C for 60 min and 70 °C for 10 min to inactivate the enzyme. Real-time PCR was performed on a QuantStudio 5 system using SYBR Green Premix Ex Taq (Takara) in a total volume of 20 µL containing 2 µL cDNA, 0.5 µL each of forward and reverse primers (10 pmol·µL^−1^), 10 µL SYBR Green Premix, and 7 µL sterile water. The amplification protocol consisted of initial denaturation at 94 °C for 2 min, followed by 45 cycles of 94 °C for 5 s and 60 °C for 30 s, with a final extension at 72 °C for 10 min. Melting curve analysis was performed to verify amplification specificity. Relative mRNA expression was calculated using the 2^−ΔΔCt^ method normalized to GAPDH. Primer sequences are listed in Table 1.

### 4.10. ELISA

Cardiac tissue was homogenized in PBS with protease inhibitors. Levels of pyruvate, acetyl-CoA, acetylcholine, and glycerophosphocholine were measured using commercial ELISA kits (Nanjing Jiancheng, Nanjing, China). Protein concentration was determined by BCA assay.

### 4.11. Statistical Analysis

All continuous variables were tested for normality using the Shapiro-Wilk test (SPSS Statistics 28.0). Normally distributed data are presented as mean ± standard deviation (SD). Statistical analysis was performed using GraphPad Prism 8. Multiple-group comparisons were conducted using one-way analysis of variance (ANOVA), followed by Tukey‘s HSD post hoc test for pairwise comparisons after confirming homogeneity of variances with Levene’s test. Effect sizes were quantified by partial eta-squared (η^2^). A value of *p* < 0.05 was considered statistically significant.

## 5. Conclusions

This integrated multi-omics study suggests that THP and BBR exert cardioprotective effects against AMI through a common mechanism of restoring glycerophospholipid homeostasis and suppressing inflammation. However, their functional roles diverge: BBR preferentially reduces pro-inflammatory lipid species (e.g., LPC 16:0, CerP 47:2), whereas THP shows a greater propensity to restore structural membrane lipids (e.g., PC 38:5, PC 40:9, SM 49:6). Notably, the differential regulation of AChE mRNA—upregulated by THP but not by BBR—serves as a molecular discriminant between their actions. These findings provide a multi-omics framework for understanding the “same disease, different treatments” paradigm and highlight the translational potential of these alkaloids as adjunctive agents.

## Figures and Tables

**Table 1:** Primer Information.

Primer Sequences	Primer	Products
ChAT Forward primer	5′-ACCTTCTGCATCTCCAGCTT-3′	176 bp
ChAT Reverse Primer	5′-AGATTGCTTGGCTTGGTTGG-3′	
CHKα Forward Primer	5′-CAGAGTTCAGCAACTGCACA-3′	152 bp
CHKα Reverse Primer	5′-TCTCCTGGCCTTCCAACAAT-3′	
PDHA1 Forward primer	5′-CATGGCTTCACCTTCACTCG-3′	91 bp
PDHA1 Reverse Primer	5′-CGCCTTTCCCTTTAGCACAA-3′	
PEMT Forward primer	5′-CATGGCTTCACCTTCACTCG-3′	143 bp
PEMT Reverse Primer	5′-TAAGTGCAGTTGTCGGGCTC-3′	
AChE Forward primer	5′-GAGATGCCATGAGTGCAGTG-3′	119 bp
AChE Reverse primer	5′-GGCACGGTGTTCAAAGATGT-3′	
GAPDH Forward primer	5′-GGTGAAGGTCGGTGTGAACG-3′	233 bp
GAPDH Reverse primer	5′-CTCGCTCCTGGAAGATGGTG-3′	

## Data Availability

The raw data supporting the conclusions of this article will be made available by the authors upon request.

## Supplementary Materials

The following supporting information can be downloaded at: https://www.mdpi.com/article/10.3390/ijms27167484/s1.

## Abbreviations

AChE	Acetylcholinesterase
AFADESI-MSI	Air Flow-Assisted Desorption Electrospray Ionization Mass Spectrometry Imaging
AMI	Acute Myocardial Infarction
AST	Aspartate Aminotransferase
BBR	Berberine
BCA	Bicinchoninic Acid
CerPE	Ceramide Phosphoethanolamine
ChAT	Choline Acetyltransferase
CHKα	Choline Kinase Alpha
CK	Creatine Kinase
CK-MB	Creatine Kinase Myocardial Band
CMC-Na	Sodium Carboxymethyl Cellulose
DAPI	4′,6-Diamidino-2-Phenylindole
ELISA	Enzyme-Linked Immunosorbent Assay
GAPDH	Glyceraldehyde-3-Phosphate Dehydrogenase
H&E	Hematoxylin and Eosin
HBDH	α-Hydroxybutyrate Dehydrogenase
HMDB	Human Metabolome Database
HW/BW	Heart Weight to Body Weight Ratio
HW/TL	Heart Weight to Tibia Length Ratio
IL-1β	Interleukin-1 Beta
IL-6	Interleukin-6
KEGG	Kyoto Encyclopedia of Genes and Genomes
LAD	Left Anterior Descending Coronary Artery
LDH	Lactate Dehydrogenase
LPC	Lysophosphatidylcholine
LVAW;d	Left Ventricular Anterior Wall Thickness at End-Diastole
LVAW;s	Left Ventricular Anterior Wall Thickness at End-Systole
LVEF	Left Ventricular Ejection Fraction
LVFS	Left Ventricular Fractional Shortening
LVVol;d	Left Ventricular Volume at End-Diastole
LVVol;s	Left Ventricular Volume at End-Systole
MSI	Mass Spectrometry Imaging
OPLS-DA	Orthogonal Partial Least Squares Discriminant Analysis
PC	Phosphatidylcholine
PCA	Principal Component Analysis
PDHA1	Pyruvate Dehydrogenase E1 Subunit Alpha 1
PEMT	Phosphatidylethanolamine N-Methyltransferase
PLS-DA	Partial Least Squares Discriminant Analysis
RT-PCR	Real-Time Polymerase Chain Reaction
SM	Sphingomyelin
THP	Tetrahydropalmatine
TNF-α	Tumor Necrosis Factor Alpha
UHPLC-Q-Exactive Orbitrap MS	Ultra-High-Performance Liquid Chromatography Quadrupole Exactive Orbitrap Mass Spectrometry
VIP	Variable Importance in Projection
