# Supplementary material for: Spatial Metabolomics Reveals the Common and Compound-Specific Pharmacological Mechanisms of Two Alkaloids Against Infarcted Myocardium
Source: Int J Mol Sci. 2026 Aug 21;27(16):7484. doi: 10.3390/ijms27167484 (PMC13513342; doi:10.3390/ijms27167484)
Supplement: Supplementary file 1 [file ijms-27-07484-s001.zip › ijms-4477016-supplementary.pdf]

Supporting Information

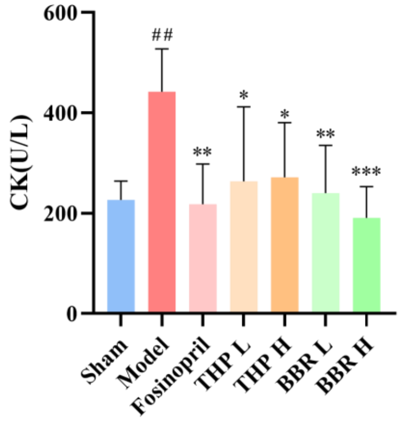

Figure S1. Serum myocardial enzyme profile CK expression levels.

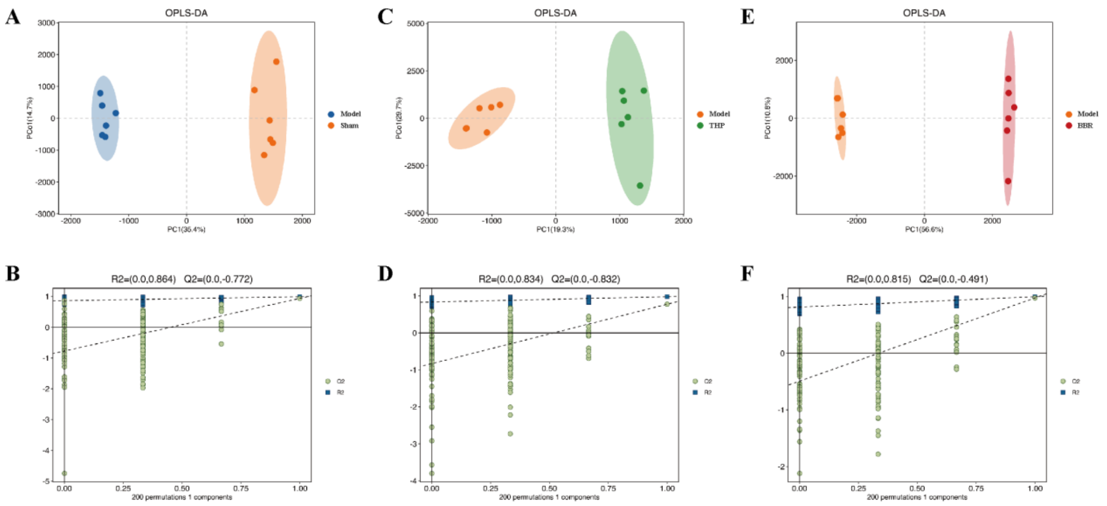

Figure S2. Serum Sample Metabolomics OPLS-DA and RPT Score Plot. (A) OPLS-DA (Sham VS Model). (B) RPT (Sham VS Model). (C) OPLS-DA (THP VS Model). (D) RPT (THP VS Model). (E) OPLS-DA (BBR VS Model). (F) RPT (BBR VS Model).

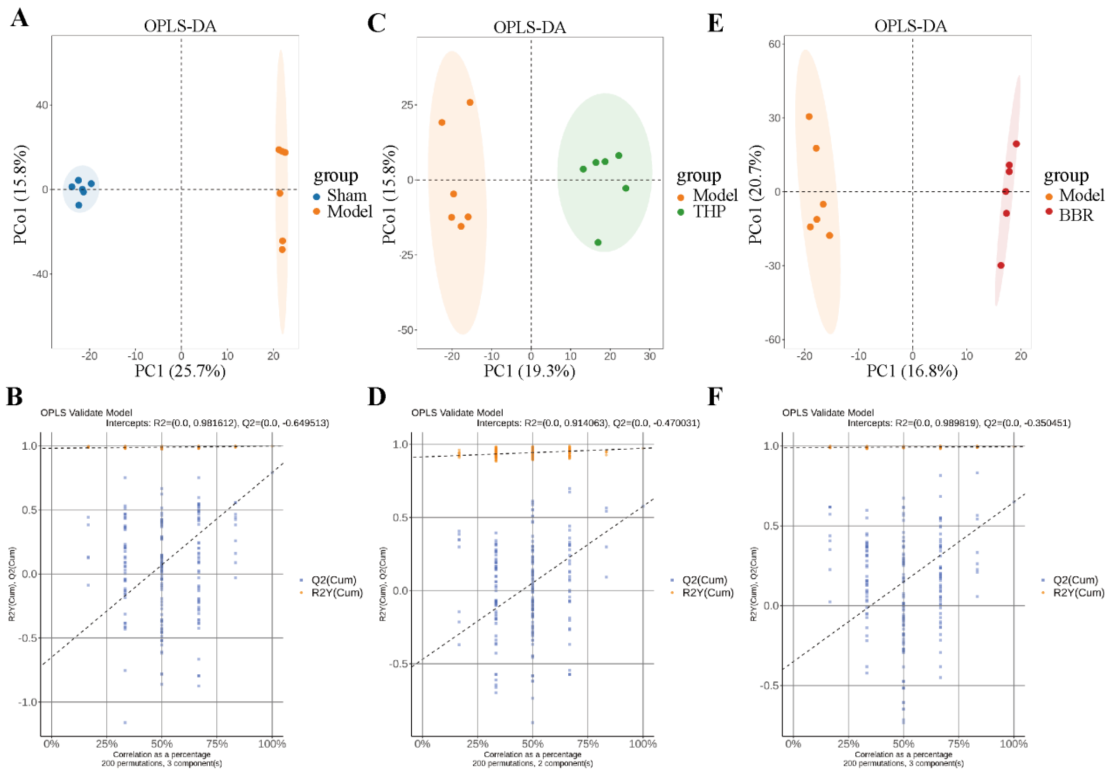

Figure S3. Lipidomics OPLS-DA and RPT Score Plots of Cardiac Tissue Samples. (A) OPLS-DA (Sham VS Model). (B) RPT (Sham VS Model). (C) OPLS-DA (THP VS Model). (D) RPT (THP VS Model). (E) OPLS-DA (BBR VS Model). (F) RPT (BBR VS Model).

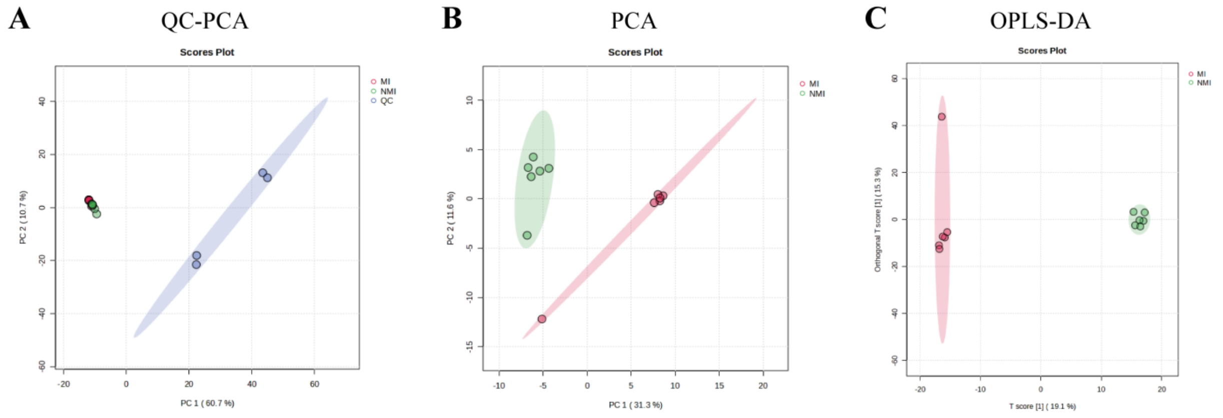

Figure S4. Spatial metabolomics PCA and OPLS-DA maps of myocardial infarction versus non-infarction regions in cardiac tissue samples (MI VS NMI)
